# Designing tuberculosis vaccine efficacy trials – lessons from recent studies

**DOI:** 10.1080/14760584.2019.1593143

**Published:** 2019-03-20

**Authors:** Ann M. Ginsberg

**Affiliations:** Clinical Development, International AIDS Vaccine Initiative, New York, NY, USA

**Keywords:** BCG revaccination, correlate of protection, efficacy trial, H4:IC31®, incipient TB, M72/AS01_E_, prevention of disease (POD), prevention of infection (POI), prevention of recurrence (POR), tuberculosis

## Abstract

**Introduction**: Tuberculosis (TB) is the leading infectious killer globally and new TB vaccines will be crucial to ending the epidemic. Since the introduction in 1921 of the only currently licensed TB vaccine, BCG, very few novel vaccine candidates or strategies have advanced into clinical efficacy trials.

**Areas covered**: Recently, however, two TB vaccine efficacy trials with novel designs have reported positive results and are now driving new momentum in the field. They are the first Prevention of Infection trial, evaluating the H4:IC31 candidate or BCG revaccination in high-risk adolescents and a Prevention of Disease trial evaluating the M72/AS01_E_ candidate in *M.tuberculosis*-infected, healthy adults. These trials are briefly reviewed, and lessons learned are proposed to help inform the design of future efficacy trials. The references cited were chosen by the author based on PubMed searches to provide context for the opinions expressed in this Perspective article.

**Expert opinion**: The opportunities created by these two trials for gaining critically important knowledge are game-changing for TB vaccine development. Their results clearly establish feasibility in the relatively near term of developing novel, effective vaccines that could be crucial to ending the TB epidemic.

## 1. Introduction

### 1.1. Methodology

The references cited below were chosen by the author based on literature searches of the PubMed database (National Library of Medicine, U.S. National Institutes of Health) to provide context for the opinions expressed in this Perspective article and sources of further information for the interested reader.

### 1.2. Scope of the global TB epidemic and urgent need for effective vaccines

TB continues to loom large on the global stage, as a public health emergency [1,2]. In 2017, TB was one of the top ten causes of death and the leading infectious killer due to a single pathogen, causing approximately 10 million new cases and killing approximately 1.6 million people [3]. The World Health Organization (WHO) End TB Strategy and the Stop TB Partnership’s Global Plan to End TB both highlight the crucial role effective pre- and post-exposure vaccines would have in ending this epidemic [2,4,5]. Modeling has demonstrated that the greatest global impact of any single new tool would result from a vaccine targeted at adolescents and adults, as these age groups are responsible for the vast majority of transmission. For example, modeling demonstrated that an adolescent/adult vaccine under most scenarios would avert more cases of TB in infants than a vaccine targeted directly at infants [6–8]. For this reason, much recent TB vaccine development has prioritized evaluating vaccine candidates for safety, immunogenicity, and efficacy in adolescent and adult populations.

### 1.3. The currently licensed TB vaccine – BCG

Bacille Calmette-Guérin (BCG), first used in humans in 1921, as an orally delivered infant vaccine [9,10], is still the only type of vaccine licensed and widely available for prevention of TB. While widely delivered intradermally under the WHO Expanded Programme on Immunization to newborns in high burden countries and moderately effective in preventing children under five years from contracting severe, extra-pulmonary TB [11], infant BCG vaccines have clearly been inadequate to control the global TB epidemic. BCGs are live, attenuated strains of *Mycobacterium bovis*, a member of the *Mycobacterium tuberculosis* (Mtb) complex and close relative of Mtb [12]. There have been several clinical efficacy trials of BCGs in a variety of settings since 1921, yielding wide-ranging results, especially in adolescents and adults. Three large efficacy trials, conducted in South India [13], Brazil [14] and Malawi [15], did not demonstrate statistically significant efficacy in adolescents and adults for prevention of TB disease, while a trial in Mtb-uninfected British adolescents demonstrated 84% efficacy in the first five years and 77% efficacy after 20 years of follow-up [16].

Hypotheses to explain such variable results have been numerous, the most predominant being influence of diverse environmental settings, including prior mycobacterial exposure. However, no explanation has yet been proven to explain the variability. A thorough review of prior BCG efficacy trials is beyond the scope of this Perspective, but they have been well reviewed elsewhere [17–19]. Another live mycobacterial vaccine candidate, *Mycobacterium microti*, showed promise similar to BCG’s in an efficacy trial conducted by the United Kingdom Medical Research Council in British adolescents starting in the 1950s [20].

### 1.4. Past TB vaccine efficacy trials

Only a very few TB vaccine candidates have advanced as far as efficacy trials for prevention of TB in recent history. These include the inactivated whole cell vaccines, *Mycobacterium indicus pranii* (also called *Mycobacterium w* [21]), *Mycobacterium obuense* (‘SRL-172’; thought at the time to be *Mycobacterium vaccae*) [22] and the virus-vectored subunit vaccine candidate, MVA85A [23]. Additionally, a randomized, controlled Phase 3 trial of *Mycobacterium vaccae* in 10,000 participants was completed in 2017 in China by Anhui Zhifei Longcom Biopharmaceutical Co., Ltd, evaluating a regimen of six injections to prevent TB disease in individuals with latent TB infection (LTBI; as determined by the tuberculin skin test), but results are not publicly available to the best of this author’s knowledge at the time of this writing (NCT01979900).

*M.indicus pranii* efficacy against TB was evaluated in the context of a leprosy prevention trial in a post-hoc analysis conducted 10–13 years post-vaccination [21]. Incidence and prevalence of TB in the trial population were determined by an active survey and retrospective analysis of the TB treatment records of the population in the intervening years. Approximately 24,000 healthy contacts of leprosy patients from the leprosy prevention trial were evaluated in this TB study. The authors report a statistically significant (1% level of significance, *Z*> 2.58) difference between the number of prevalent pulmonary TB cases requiring treatment identified in vaccinees (n = 29/69) vs. placebo recipients (n = 40/69).

The *Mycobacterium obuense* Phase 3 efficacy trial, known as the DarDar trial ([22]; NCT00052195), was conducted in HIV-infected Tanzanian adults with a CD4 count of ≥200 cells/µl and a BCG scar. A series of five injections of SRL-172 did not demonstrate efficacy in preventing the primary endpoint of ‘disseminated TB’ (P = 0.16) or a secondary endpoint of ‘probable TB’ (P = 0.46) but did demonstrate a statistically significant vaccine efficacy of ~39% (intent-to-treat population: hazard ratio of 0.61; 95% confidence interval (CI) = 0.39–0.96; P = 0.03) for prevention of a secondary endpoint of ‘definite TB’ (defined as positive by sputum smear, blood or sputum culture, or evidence of caseous necrosis on biopsy). However, SRL-172 did not have a scalable manufacturing process and so did not advance further in development. Instead, a new process was devised by Aeras in collaboration with Dartmouth College (the Sponsor of SRL-172) involving liquid growth of *Mycobacterium obuense* (grown from the same Master Cell Bank as SRL-172) instead of using growth on solid agar, and the resulting vaccine candidate, DAR-901, was evaluated in Phase 1, first time in human, safety, immunogenicity and dose-finding study ([24]; NCT02063555). DAR-901 then advanced into a Phase 2 proof-of-biological-effect trial (NCT02712424) in Tanzanian adolescents, with a primary endpoint of prevention of new Mtb infection (defined by new T-Spot TB interferon-gamma release assay (IGRA) conversion) and a secondary endpoint of new, persistent Mtb infection (defined as “new positive IGRA that is also positive on repeat ≥3 months later“; see below discussion of endpoints and prevention of infection trials). The trial is expected to be completed by the end of 2019 (see clinicaltrials.gov NCT02712424).

The subunit vaccine candidate, MVA85A, was evaluated in a phase 2b trial in BCG-vaccinated infants, but, disappointingly, did not demonstrate efficacy beyond that of BCG alone. A second MVA85A trial, ongoing at the time, was originally designed as an efficacy trial in HIV-infected, African adults. As a result of the infant trial outcome and to save scarce resources for the field, this adult trial was downsized and refocused as a safety and immunogenicity trial and therefore was no longer powered to evaluate efficacy ([25]; NCT01151189).

## 2. Results and lessons from recent TB vaccine efficacy trials

### 2.1. Recently completed TB vaccine efficacy trials

In a unique occurrence for TB vaccine development, two clinical efficacy trials published their results in 2018: a prevention of Mtb infection (POI) trial of Statens Serum Institut’s and its partner’s, Sanofi Pasteur’s, novel subunit candidate vaccine, H4:IC31®, or BCG revaccination compared to placebo in Mtb-uninfected, HIV-uninfected, healthy adolescents at high risk of Mtb infection ([26]; NCT02075203), and a prevention of active TB disease trial of GlaxoSmithKline’s candidate, M72/AS01_E_, compared to placebo in healthy, Mtb-infected adults, which recently published its primary analysis ([27]; NCT01755598). Once the final results of the second trial are published, full safety, immunogenicity, and efficacy data will be available for three vaccine candidates (H4:IC31, BCG revaccination and M72/AS01_E_) – an unprecedented treasure trove of human data for this field. In addition, and crucially, both trials had samples collected and biobanked to enable potential discovery of candidate correlates of risk and protection, an initiative which is now in planning stages in collaboration with the broader TB research community.

Main results from the Phase 2 H4:IC31/BCG revaccination POI trial, the first TB vaccine trial conducted with a POI endpoint (measured by initial IGRA conversion [primary endpoint] or sustained IGRA conversion [secondary endpoint]) conducted in the TB vaccine field, were: (1) neither vaccine met statistical significance for vaccine efficacy for the primary endpoint (lower bound of the 95% confidence interval was <0); (2) BCG revaccination demonstrated vaccine efficacy of 45.4% (p = 0.03) against sustained IGRA conversion; and (3) both vaccines were immunogenic and had acceptable safety profiles in this high-risk adolescent population [26]. Therefore, this trial demonstrated the potential for a new use of BCG (through revaccination) in protecting high-risk populations from becoming infected with Mtb. BCG has been delivered to infants approximately four billion times since its first introduction in 1921 [19], is generally safe in individuals with intact immune systems, and is inexpensive [28]. Mathematical modeling has demonstrated that BCG revaccination could be ‘highly cost-effective’, at all combinations of cost (US$1–10) and efficacy (10–80%) evaluated [29]. Therefore, further investigation as a potential public health intervention is warranted and a larger, confirmatory Phase 2b trial of BCG revaccination is in the planning stages. These results did not support a product development decision to advance H4:IC31 into further development.

Results of the primary analysis of the M72/AS01_E_ phase 2b prevention of disease (POD) trial based on at least two years of participant follow-up, conducted in healthy African adults who were already Mtb-infected at baseline, included: (1) M72/AS01_E_ met the primary endpoint of preventing liquid culture and/or Xpert MTB/RIF confirmed pulmonary TB not associated with HIV, demonstrating a vaccine efficacy of 54.0% (90% CI, 13.9 to 75.4; 95% CI, 2.9 to 78.2; p = 0.04); and (2) M72/AS01_E_ was more reactogenic than placebo, primarily attributable to its causing more local injection site pain and swelling and flu-like symptoms, but had an acceptable safety profile, with the two groups demonstrating similar rates of serious adverse events, potential immune-mediated diseases, and deaths, with none of the deaths being attributable to vaccine. The trial is still ongoing until participants complete three years of follow-up; final results are anticipated in 2019. These results are the first statistically significant indication of a subunit vaccine’s efficacy (in this case, consisting of just two Mtb proteins and an adjuvant system) being able to protect even partially against TB disease and the first evidence of any vaccine being able to protect already Mtb-infected individuals from advancing to active disease.

Given the value to the TB vaccine field of these two trials, it is important to review key issues considered in their design and attempt to extract lessons that could be helpful in designing future TB vaccine efficacy trials.

### 2.2. Key efficacy trial design considerations

Three factors were key to the design of these recent efficacy trials: (1) pressure of limited available resources, (2) choice of efficacy endpoints and (3) choice of trial target population. The second and third factors stemmed, in large part, from the first: an effort to decrease the resources needed for TB vaccine efficacy trials catalyzed the development of novel trial designs with innovative, rigorously defined endpoints and ‘high risk’ populations.

#### 2.2.1. Pressure of limited available resources

In the wake of the MVA85A efficacy trial results, there was an elevated sense of disappointment in the global health research and funding communities and questioning of the feasibility of developing effective TB vaccines in the absence of greater fundamental understanding of the human host–pathogen relationship (see, for example [30],). This, in turn, led to a general shift in focus and funding to more basic research and away from advancing TB vaccine candidates into the clinic and especially into resource-intensive, efficacy trials. As a result, decreasing efficacy trial costs was the original catalyst for developing the novel trial designs referred to below and a significant consideration in the design of both recent trials. Two key drivers of trial costs are sample size (number of participants) and trial duration, and approaches to decreasing each were deliberated extensively in the process of creating designs for future efficacy trials.

#### 2.2.2. Choice of efficacy endpoints

Choice of a high incidence endpoint can help to decrease both sample size and duration of follow-up. For the H4:IC31 and BCG revaccination trial, a novel POI trial was designed by the trial partners (Aeras and Sanofi Pasteur) because it was known that the incidence of Mtb infection is approximately 8–10-fold higher than the rate of active TB disease in a given population [31,32], and clinical trial sites could be identified that have a very high annual incidence of infection (force of infection), including the South African TB Vaccine Initiative site and the Desmond Tutu HIV Vaccine Foundation Emavundleni Clinical Research Site in the Western Cape region of South Africa [33,34]. Identifying a measurable endpoint (in this case, an endpoint based on conversion of an IGRA) that occurs in the chosen population with very high incidence enabled the sample size to be relatively small and the trial duration to be shorter compared to a trial design based on the traditional TB vaccine trial efficacy endpoint of POD in the general, healthy population. A relatively small sample size and shorter trial duration, in turn, reduced trial costs compared to the classically utilized prevention of active TB disease in a general, healthy population trial design.

A POI endpoint also had the advantage of creating an opportunity to evaluate for the first time in a prospective, randomized trial whether BCG could prevent Mtb infection in previously uninfected adolescents. Although BCG has been ineffective globally in adequately controlling TB in adolescents and adults, there were several natural history and retrospective human cohort studies providing evidence that BCG may protect against infection (reviewed in [17,31]; and see [35]), but a prospective, randomized trial had not been conducted in adolescents or adults to confirm these interesting results. A complexity of relying on prevention of infection to test a TB vaccine’s efficacy is that even if vaccine efficacy were demonstrated for prevention of infection, this meaningful biologic activity might not translate into an ability of the vaccine to prevent disease. This subtlety stems from the natural history of TB infection and disease, in which only approximately 10% of individuals who get infected by Mtb ultimately develop TB disease. The remaining 90% are able to clear or control the infection, never becoming clinically symptomatic and generally not being a source of transmission (hence being said to have ‘latent TB infection’, or LTBI, diagnosed only by the tuberculin skin test or an IGRA) [36,37]. As a result, if a vaccine had 90% or less vaccine efficacy against infection, it theoretically could be protecting only individuals who would have controlled the infection even in the absence of vaccination. If that theoretical possibility were true, the vaccine would not prevent any cases of active disease. On the other hand, if the vaccine is preventing infection in individuals who, without vaccination, would go on to develop active TB disease, it could potentially have significant public health impact in some settings, as has been demonstrated by mathematical modeling [8]. Therefore, demonstration of POI must be followed by a POD trial before one can conclude that the vaccine could have a meaningful effect in controlling the TB epidemic. This concern about the potential for minimal impact from a vaccine that works only by preventing infection, along with the fact that Mtb infection is diagnosed by an imperfect laboratory test (whether skin test or IGRA), makes it unlikely that demonstrated POI in the absence of demonstrated POD would be deemed a licensable endpoint by a stringent regulatory authority or adequate to support implementation by a public health authority (except perhaps in the context of infant BCG replacement where POD trials might not be feasible). The POI endpoint was therefore chosen for the H4:IC31 and BCG revaccination trial as the basis for an early ‘proof of biological effect’ study to help support product development decision-making for the H4 candidate vaccine at relatively low cost, but expecting that, in and of itself, this trial would not be the basis for licensure. The intention, if H4:IC31 or BCG revaccination met pre-set POI efficacy and safety criteria, was to advance to a POD trial.

A subsequent issue raised by the decision to conduct a POI trial was whether the primary efficacy endpoint would be prevention of initial IGRA conversion from negative to positive or whether it should be prevention of sustained IGRA conversion (that is once an IGRA converted to positive whether it remained positive on repeated testing for at least six months). The theoretical question was whether BCG or the novel subunit vaccine would work by preventing Mtb from becoming established enough to prime T cells in the lymph nodes after entering the respiratory tract (prevention of initial IGRA conversion) or whether the bacilli would be breathed in, traffic to lymphoid tissue, and prime T cells (immune sensitization reflected in initial IGRA conversion), but then be adequately controlled by the vaccine-induced host immune response to be cleared or at least have bacillary replication controlled enough in the subsequent few months that the IGRA reverted to negative, that is below the manufacturer’s cut-off for positivity of 0.35 IU/ml (prevention of sustained IGRA conversion). This question was thoroughly debated by the vaccine and trial sponsors, trial site investigators, immunologists and biostatisticians involved in the protocol design. In the end, a key reason for choosing prevention of initial IGRA conversion as the primary endpoint was there was more robust epidemiology data to support sample size and study powering assumptions for prevention of initial conversion than for prevention of sustained conversion, particularly in the absence of a standard definition of ‘sustained conversion’ from previous studies. Well-defined rates of IGRA reversion post-initial conversion in the study population were not available at the time of study design.

Without an identified correlate of protection from Mtb infection or TB disease, it was clear in designing both the POI and Phase 2b POD trials that efficacy would have to be evaluated based on clinically relevant efficacy endpoints. Trial designs with novel efficacy endpoints had been proposed [31,32] but not yet implemented. These two trials were among the very first opportunities to do so. The classically measured endpoint in previous TB vaccine efficacy trials is a prevention of disease (POD) endpoint, with varying degrees of rigor used to define the disease. These endpoint definitions have ranged from a clinically determined endpoint to microbiologically defined endpoints based on microscopic diagnosis (including sputum smear or other biologic fluid or tissue evidence of acid-fast bacilli with or without x-ray diagnosis of pulmonary TB), solid or liquid culture-confirmed Mtb and/or nucleic acid amplification-based confirmation, using Xpert MTB/RIF [38] or another rapid diagnostic test. These two trials were designed with as rigorously defined, laboratory-confirmed endpoints as feasible. The POI endpoint is facilitated by the relatively recent availability of IGRAs (including the QuantiFERON TB Gold-in-tube® or TB Plus® and T-Spot TB® assays) that are more specific for Mtb infection than the tuberculin skin test [39]. A rigorous, microbiologically confirmed POD endpoint can be based on diagnosis with a validated, automated liquid culture system such as the MGIT® and/or a validated nucleic acid amplification test such as the Xpert MTB/RIF®.

A third novel efficacy trial design proposed for TB is a specific sub-type of a POD trial in a high-risk population – one that evaluates a vaccine’s efficacy in preventing recurrent TB following ‘curative’ TB treatment (Prevention of Recurrence or POR trial). Recurrent TB diagnosis should again be based on rigorous microbiologic confirmation. Additionally, if the study is adequately powered, the distinction of recurrence due to endogenous reactivation of the initial infecting strain (‘relapse’) from recurrence due to reinfection with a different Mtb strain (for example, by whole genome sequencing) could provide key insight into the vaccine’s mechanism of action.

#### 2.2.3. Choice of target population

Choice of the trial populations in which to test the vaccine candidates was of course closely intertwined with the choice of efficacy endpoints. Adolescents in the Western Cape of South Africa were chosen for the POI trial largely because of the very high force of infection experienced by this population. Similarly, for the M72 POD trial, the chosen population was Mtb-infected (IGRA positive) adults in three African countries (Kenya, Zambia and South Africa), estimated to have an average annual incidence of TB at the selected trial sites of approximately 550 per 100,000 population. This incidence is well above the global average annual TB incidence of 133 per 100,000 population [3] and presumably higher than that in IGRA-negative individuals in the same populations [40]. As with the POI trial, choosing this clearly defined and epidemiologically important population at high risk for the primary efficacy endpoint was driven in part by a desire to decrease the required sample size as a means for limiting trial costs.

### 2.3. Lessons learned concerning TB vaccine efficacy trial design

The positive results from these two recent ‘proof of concept’ efficacy trials and the opportunities they have created for gaining critically important knowledge are truly game-changing for TB vaccine development. In addition, they provide crucial data to support further evaluation of BCG revaccination as a potentially impactful public health intervention and of M72/AS01_E_ to protect against TB disease. Perhaps most importantly, these results clearly establish the feasibility in the relatively near term of developing novel, effective TB vaccines to help control the epidemic.

Lesson 1 – POI trials of TB vaccines are a feasible and relatively low-cost approach to demonstrating a meaningful biological vaccine effect but carry associated risks

The two recent efficacy trials have already led to important learnings related to choosing the overall trial objective. A key choice is whether to design a trial that assesses, for example, a vaccine’s ability to prevent Mtb infection (POI trial), active TB disease (POD trial) or disease recurrence following treatment cure (POR trial). This decision will need to take into account multiple strategic considerations, including the candidate’s target product profile, stage of development and overall product development plan, keeping the ultimate licensure and access requirements for the vaccine in mind.

The H4/BCG revaccination trial demonstrated for the first time that a POI trial design is feasible for TB vaccines and can be utilized to demonstrate that the vaccine has a clinically relevant biological effect. However, there are risks associated with using a POI design for a vaccine candidate’s first efficacy trial in that: (1) a vaccine may prevent active disease but not initial or sustained infection – so a good candidate could be mistakenly terminated if efficacy is not seen against infection but would have been demonstrated if the trial endpoint were active disease; (2) demonstration of POI efficacy must translate into subsequent POD for the vaccine to have substantial public health benefit, so a successful POI trial will need to be followed by a more resource-intensive POD trial, and (3) to further decrease risk, a modestly sized POI trial (like the completed H4/BCG revaccination trial) may be followed first by a larger, confirmatory POI trial before being advanced to a POD trial. Conduct of a POI trial to support early product development decision-making saves resources up-front when risk is highest, but in the long run may require more resources and extend development timelines compared to conducting a Phase 2b POD trial as a first efficacy trial.

Once a POI trial design is chosen, the primary endpoint deserves careful further consideration. Designers of future POI trials may be wise to use prevention of sustained IGRA conversion as the primary efficacy endpoint rather than prevention of initial IGRA conversion. Powering assumptions related to IGRA reversion rates and timing of IGRA testing can now be based on data from the first POI trial (if the same definition of sustained conversion is used and the study is conducted in similar populations; see also Lesson 5, below) to make sample size calculations and to refine protocol design with respect to frequency of IGRA testing, helping to maximize the opportunity to demonstrate efficacy and realize cost-efficiency.

Lesson 2 – Use rigorously defined efficacy endpoints for proof of concept trials

The BCG revaccination POI trial’s efficacy endpoints relied on the QuantiFERON-TB Gold-in-Tube IGRA. Robust results were aided by using an assay protocol within the confines of the manufacturer’s recommended protocol, but applying even more stringent assay conditions and standard operating procedures, which were established through a careful evaluation process of multiple conditions to try to decrease ‘indeterminate’ results and enhance reproducibility [41]. Alternate thresholds for assay positivity formed the basis for several exploratory analyses in the POI trial that are providing insights into correlation between quantitative assay results and risk of sustained infection [26].

The M72 Phase 2b trial made clear that basing the first and second case definitions on rigorously defined microbiological confirmation of Mtb in sputum specimens from pre-TB treatment, HIV-uninfected individuals with clinical suspicion of pulmonary TB was important for demonstrating statistically significant vaccine efficacy [27]. An even more stringent microbiologic definition of pulmonary TB than the trial’s first case definition, requiring at least two positive culture and/or nucleic acid amplification test results across three distinct sputum specimens (see the sensitivity analysis performed on the first case definition [27],), would have resulted in a higher observed point estimate for vaccine efficacy for the primary endpoint (70% vs. 54%) but would have required a larger sample size to achieve the same power as the current design, all other assumptions being equal. The trial results also demonstrated that using both automated liquid culture and a nucleic acid amplification-based test, in this case the Xpert MTB/RIF test, was valuable in that liquid culture had higher sensitivity, consistent with others’ findings [38], while Xpert MTB/RIF, by providing much more rapid turnaround of results, facilitated both rapid clinical care decisions and baseline screening to exclude those with Mtb-positive sputum. Use of liquid culture at baseline to exclude active TB was judged not feasible given that Mtb culture requires up to 42 days to rule out a positive result.

Lesson 3 – Choice of exact trial population benefits from taking multiple factors into account

These two trials have also demonstrated the value of choosing the trial population carefully based on a variety of factors, including expected vaccine mechanism of action, the primary trial endpoint, the candidate’s target product profile (e.g., infant vaccination or adolescent/adult vaccination) and the stage in the clinical development plan (proof of concept vs. Phase 3 licensure trial). Choosing to enroll only a population at high risk of acquiring the primary efficacy endpoint can help reduce costs and duration of a proof of concept trial but may not be appropriate in a Phase 3 trial to support licensure if the ultimate development goal is vaccination of a broader population, as would be likely for new TB prevention vaccines. IGRA-negative individuals were not enrolled in the M72 Phase 2b trial largely because doing so would have substantially increased trial size and, therefore, costs; that decision came with understood limitations on the ability to draw conclusions about the vaccine’s ultimate potential efficacy in the broader population and left this question to be answered in later studies.

Lesson 4 – Site-specific epidemiology data is critical

Once the general target population is chosen, choice of trial site(s) should include, amongst other important operational factors, whether there is adequate local epidemiology data in the specific population to support site selection and enable robust trial size calculations [42]. The value of having relevant site-specific epidemiology data, gathered using the same endpoints as will be employed in the clinical trial, is not unique to the design of TB vaccine trials, of course. However, the frequent lack of local Mtb infection and disease prevalence and incidence data, particularly based on these rigorous definitions, which are often different from those employed by the national TB control program, makes it worth reiterating, so that trial sponsors and their collaborators can plan for the necessary epidemiology studies in advance of finalizing trial design and in enough time to minimize delay to trial start. The takeaway from recent trials is that incurring some delay to acquire these data is likely to be a worthwhile investment.

Lesson 5: Duration of a ‘wash-out’ period can have a critical impact on the observed vaccine efficacy

Although also not unique to TB vaccine trial designs, the lack of detailed understanding of most TB vaccine candidates’ mechanisms of action can make the choice of how long to wait before including endpoints in the primary analysis (‘wash-out’ period post-vaccination) particularly challenging. The goal is to exclude endpoints that occur so soon after vaccination that it is not reasonable to assume the vaccine could have prevented them, while including all endpoints that the vaccine should be able to prevent. While an 84-day wash-out from day of first vaccination in the POI trial [26] appears to have been successful in eliminating most or all subjects who were already Mtb-infected at baseline but had not yet converted their IGRA, it is possible that the one-month post-vaccine dose 2 wash-out period in the M72 phase 2b trial [27] was not adequate to exclude all participants who were already well on the path to developing active TB (i.e., had clinically asymptomatic, incipient TB) at the time of enrollment [43]. It may not be feasible for a vaccine to protect those who already have incipient TB at baseline and therefore, the potential for such individuals to have been enrolled in the trial could explain why the two vaccination groups (M72 and placebo) had similar rates of TB in the first approximately nine to 12 months of post-wash-out follow-up, only after which the vaccine efficacy became apparent [27]. Future proof of concept trials may be able to exclude those with incipient TB through the use of a biosignature marker [44–46]. The duration of the wash-out period may also vary with the type of vaccine candidate being tested and its mechanism of action – for example, maximal immune responses to BCG and other whole-cell vaccines may take over two months to develop, whereas development of maximal immune responses to adjuvanted proteins or other subunit vaccines may require only two to four weeks.

Lesson 6 – Human efficacy trials can provide previously unavailable insights into vaccine mechanism of action

As noted above, in the POI trial, BCG revaccination demonstrated statistically significant vaccine efficacy against sustained IGRA conversion but not against initial IGRA conversion [26]. This result suggests that BCG revaccination does not entirely prevent Mtb infection, but rather even in vaccinated individuals, bacilli enter the airways and traffic to local lymph nodes where they activate CD4 + T cells, which in turn secrete interferon gamma upon re-stimulation with vaccine-specific Mtb antigens during an IGRA. However, BCG revaccination increases bacterial clearance and/or control such that repeat IGRA testing within six months post-initial IGRA conversion will demonstrate an increased percentage of individuals who have reverted their IGRA result to negative compared to those vaccinated with placebo. A role for trained innate immunity in this process of bacillary control or clearance would be of interest to explore given recently reported results in this field [47–51].

In the M72 Phase 2b trial [27], a sub-group analysis (for which the study was not powered and therefore should be interpreted with caution) indicated that individuals 18–25 years old may be protected at a higher rate by the vaccine than those >25–50 years old. Within the placebo arm, the younger group also experienced twice the incidence of TB (case definition 1) on average as the older group (0.8 vs. 0.4% per year [27]), which is consistent with the hypothetical possibility that more frequent exposure post-vaccination boosts vaccine efficacy. Also, consistent with this hypothesis, a Kaplan-Meier analysis of vaccine efficacy [27] suggests efficacy may increase with time from vaccination during the first two years of follow-up. Results of the Kaplan-Meier analyses of both the BCG revaccination and M72/AS01_E_ trial endpoints are consistent with the hypothetical possibility that repeated, post-vaccination exposure to Mtb boosts vaccine efficacy. This hypothesis needs further evaluation in future studies. Another possible explanation for potentially higher efficacy in the younger population in the M72/AS01_E_ trial is that, on average, the younger participants are closer to the time of their initial Mtb infection (which occurs mainly during adolescence in the trial population); as a result, their infections may be more susceptible to control or clearance by vaccine-induced immune responses.

Further exploration of these hypotheses ultimately may lead to important insights into these vaccines’ mechanisms of action. The M72 Phase 2b trial could have contributed further to understanding that vaccine’s mechanism of action if IGRA testing post-vaccination had been included to assess whether the vaccine increased IGRA reversion rates in this already infected adult population. Increased reversion rates would have suggested the vaccine had an effect on clearing or controlling the bacilli and preventing sustained infection in addition to preventing active disease.

Lesson 7 – Collecting biospecimens for correlate analyses should be considered a fundamental aspect of TB vaccine efficacy trials

The two efficacy trials that are the focus of this article have helped to emphasize that the drive to minimize trial costs must be weighed against the consequent limitations incurred on the robustness of the resulting data (e.g., trial power and level of type 1 error, applicability of data to a broader population, ability to draw conclusions from sub-group analyses – to name just a few of the trade-offs). One casualty of overly restrictive budgets in some past and current TB vaccine efficacy trials has been the collection of biospecimens for subsequent correlate discovery and validation. The two trials discussed here and their associated biospecimen collections are supporting initial discovery efforts for correlates of risk of and protection from sustained Mtb infection and active TB disease. Identification and validation of such correlates would enable vastly more streamlined and less costly TB vaccine development in the future, as endpoints could be based on a marker or biosignature that reflects a vaccine-induced, protective host immune response, likely measured relatively soon after vaccination. Such a correlate or biomarker of protection could markedly decrease both the long follow-up times currently needed to accrue adequate numbers of clinical endpoints and decrease the trial sample size necessary to achieve the desired power to detect a specified vaccine efficacy. Given the extraordinary value correlates of protection could provide for TB vaccine development at this point in history, it would be an incalculable loss to the TB field to conduct an efficacy trial without collecting the relevant specimens, of course under appropriate participant informed consent and independent ethical review.

Lesson 8 – Clinical trials, including efficacy trials, should be conducted in parallel with basic and preclinical TB research

Perhaps the lesson demonstrated most clearly by these two trials is the value of conducting empiric product development, including clinical efficacy trials, *in parallel with* basic research and discovery/preclinical development. Both trials were greeted with substantial levels of pessimism and skepticism as to their eventual merit before their results were known. However, the many insights gained from these trials clarify that only with human efficacy results and more upstream research progressing in parallel and informing each other in a virtuous cycle can researchers and vaccine developers base decision-making on the most relevant data and maximize progress.

## 3. Conclusion

The Prevention of Infection trial of H4:IC31 and the Prevention of Disease trial of M72/AS01_E_ discussed above not only support continued evaluation of BCG revaccination and M72/AS01_E,_ respectively, but provide insights and learnings that can help advance TB vaccine development. They demonstrate the importance to progress in this field of conducting somewhat empiric human testing, including efficacy trials, of promising candidates in parallel with continued basic and translational preclinical research.

## 4. Expert opinion

TB vaccine research and development is at an exciting juncture. Important progress has recently been made with the positive results of the two recent efficacy trials discussed here and in basic science and preclinical development of TB vaccines (reviewed in [52,53]). There is the potential to discover candidate correlates of vaccine-induced protection from TB and sustained Mtb infection based on interrogating the biorepositories created during the M72 POD trial and H4/BCG revaccination POI trial, respectively.

In the next two to five years, additional efficacy results should be reported from up to six trials, including five in adolescents and/or adults: a POI trial of DAR-901 (NCT02712424), POR trials of VPM1002 (NCT03152903), ID93 (IDRI trial TBVPX-204 [54]) and H56:IC31 (NCT03512249) and the previously mentioned Phase 3 *M.vaccae* trial in China (NCT01979900), and one Phase 3 trial of VPM1002 compared to BCG in South African infants [55]. In addition to informing the development of these candidates and providing more lessons for clinical trial design and conduct, this profusion of human data must be interrogated for insights about vaccine mechanism of action, predictive ability of preclinical models and correlates of risk and vaccine-induced protection against Mtb infection and TB disease.

At least two controlled human infection models of TB, also referred to as human challenge models, are under development. If demonstrated to be safe and predictive of vaccine efficacy in humans, a human challenge model could provide an approach to rapid triaging and optimizing of novel vaccine candidates, with or without the availability of correlate(s) of protection. A consortium of investigators, led by Eric Rubin and Sarah Fortune at Harvard and currently funded by the Bill and Melinda Gates Foundation, is developing a controlled human infection model for TB based on attenuation of Mtb, expression of a quantifiable marker system and infection by either aerosol or intradermal delivery. A second design for a human challenge model is being developed by Helen McShane and colleagues at Oxford University based on a challenge with BCG and intradermal delivery [56]. Key questions to be resolved for these models are how well immune responses induced by BCG or an attenuated Mtb mimic those necessary for protection from circulating, clinical strains of Mtb and whether intradermal challenge adequately mimics the natural, aerosol route of human infection in TB.

The opportunities created by the two recently published positive efficacy trials for gaining critically important knowledge are game-changing for TB vaccine development. Their results establish the feasibility in the relatively near term of developing novel, effective vaccines that could be crucial to ending the TB epidemic. In September 2018, the first ever United Nations General Assembly High Level Meeting on Tuberculosis was convened – a greatly needed expression of global political will to end the epidemic. Given the current extensive under-resourcing of TB research and development (R&D) [57,58], this meeting must now be followed by sustained national commitments and infusion of funds to capitalize on the recent scientific progress.

TB vaccine R&D is poised to make exceptional progress in the next five years, provided adequate resources. Given the number of candidates now in proof of concept studies, and the positive results seen in the two trials reported here, at least one novel vaccine candidate should have advanced into a Phase 3 licensure trial for prevention of TB disease in adolescents and adults in the general population. However, ensuring this critical milestone likely will require not only adequate Phase 3 resources, but formation of sustainable partnerships and commitments through vaccine delivery from a diversity of key stakeholders, as no one organization alone is likely to take on a complete product development and access strategy for a TB vaccine given the lack of an adequate market driver for this field.

Additionally, in the next five years, with similar commitments, the BCG revaccination POI trial results should be confirmed and extended, enabling a POD trial to have begun. Ideally, one or more candidate biomarkers will have been identified, and perhaps validated, stemming from the BCG revaccination POI trial and M72/AS01_E_ Phase 2b trial biorepositories. If so, development of vaccine candidates expected to induce the identified correlate(s) of protection will be dramatically advantaged in the future, being able to establish much more rapidly, using relatively small, short duration clinical studies, dose and regimen optimization and evaluate efficacy in a range of populations – for example, according to age, race, sex, HIV status (where the safety profile is appropriate) and geographic setting. Biomarkers that can help identify individuals at high risk of infection or disease (but not yet having incipient TB) for enrollment into efficacy trials may also be in use, having been discovered and validated utilizing the biobanked specimens from previous natural history cohort studies and efficacy trials.

Within five years, alternate routes of administration, such as mucosal and/or intravenous delivery, may be in the process of being evaluated in human efficacy studies for potentially enhanced vaccine-induced protection [59–66].

The coming half-decade should be a time of unparalleled advances in both upstream research and clinical product development for TB vaccines. Creative and robust new partnerships amongst public and private sector vaccine sponsors, researchers, funders, governments (of both low and high burden countries), advocates and affected communities will be vital to ensuring this exciting and essential progress continues not only through late-stage development and licensure, but through access and delivery to the populations in need of safe and effective TB vaccines.

## Article highlights

- TB is the leading infectious killer due to a single pathogen and one of the top ten causes of death globally. The only currently licensed TB vaccines, BCG strains, are widely delivered to newborns globally but have been inadequate for controlling the epidemic.
- New safe, effective vaccines would be crucial for ending the TB epidemic. By preventing the majority of transmission, vaccines delivered to adolescents and adults would likely have the fastest impact on preventing TB disease and death in all age groups.
- TB vaccine R&D is under-resourced in relation to TB’s public health burden. Consequently, very few novel vaccine candidates have progressed into human efficacy trials in the almost 100 years since BCG’s introduction.
- The year 2018 was a year of unprecedented progress with the announced positive results from two clinical efficacy trials: prevention of infection trial evaluating H4:IC31® and BCG revaccination and prevention of disease trial of M72/AS01_E_. Three key considerations were central to the design of both trials: (1) the pressure of limited available resources, (2) choice of efficacy endpoints and (3) choice of trial target population.
- The results and associated specimen biorepositories from these trials are the basis for a number of lessons that can impact the design of future clinical efficacy studies and inform more upstream TB vaccine research and development. The learnings derived from these two trials demonstrate the importance of empiric clinical product development, including efficacy trials, advancing in parallel with more upstream research and rational vaccine design.
- Novel, sustainable partnerships amongst diverse stakeholders will be crucial to developing and delivering effective vaccines to the populations who most need them.
